# A retrospective analysis of the risk factors for surgical site infections and long-term follow-up after transpalpebral enucleation in horses

**DOI:** 10.1186/s12917-017-1069-5

**Published:** 2017-06-02

**Authors:** Tsjester Huppes, Hanneke Hermans, Jos M. Ensink

**Affiliations:** Department of Equine Sciences, Faculty of Veterinary Medicine, Utrecht University, Yalelaan 114, 3584 CM Utrecht, Netherlands

**Keywords:** Horse, Transpalpebral, Enucleation, Risk factors, Surgical site infection

## Abstract

**Background:**

Implants are often used to improve the cosmetic appearance of horses after enucleation of the eye. When surgical site infection (SSI) occurs, the implant will almost always be lost. The aim of this study is to collect data on the risk factors for SSIs and report long-term follow-up (cosmetic results and return to work) after transpalpebral enucleations. In this retrospective study, records of horses undergoing transpalpebral enucleation were reviewed (2007–2014) and telephone interviews were used to obtain long term follow-up. The potential risk factors for SSIs (indication for enucleation, use of an implant, standing procedures, duration of surgery, opening of the conjunctival sac and prolonged use of antimicrobials) were analysed for their association with the outcome measure ‘SSI’ vs ‘no SSI’ by multivariable binary logistic regression testing. Indications for enucleation were grouped as follows: Group 1 (clean) included equine recurrent uveitis, too small or too large globes, and intraocular tumours, Group 2 (non-clean) included corneal perforation/rupture and infected ulcers and Group 3 (tumour) included extraocular tumours.

**Results:**

One hundred and seven cases of enucleation were evaluated. An implant was used in 49 horses. The overall number of SSIs was 8 (7.5%). Multivariable logistic regression testing showed implants (OR 7.5, *P* = 0.04) and standing procedures (OR 12.1; *P* = 0.03) were significantly associated with the percentage of SSIs and increased the risk of SSI. The eyes of horses in Groups 2 and 3 trended towards a larger risk for developing SSIs (OR 4.9; *P* = 0.09 and OR 5.9; *P* = 0.1, respectively). Prolonged use of antimicrobials, long surgery times and the opening of the conjunctival sac during dissection did not show significant associations with SSI risk.

**Conclusions:**

The risk of SSI after enucleation is low in clean eyes and when no implant is used. Placing an implant or performing a standing enucleation significantly increases the risk of SSIs. Although implants can be used for eyes that fall into Groups 2 and 3, 17% of the horses in these two groups developed an SSI leading to loss of the implant.

## Background

Enucleation is a commonly performed orbital surgery, in which the palpebral margins, nictitating membrane and its associated glands, conjunctiva and globe are removed. It is indicated in eyes that are painful and non-visual. It can also be indicated in eyes that still have visual function if they have a poor long term prognosis [1]. For simple enucleations, a transpalpebral or subconjunctival approach with primary closure of the eyelids can be used. Although preoperative flushing of the cornea and upper and lower fornices of the conjunctiva with povidone-iodine preparations significantly decrease the conjunctival flora [2, 3], in cases where sepsis or neoplasia involves the corneoconjunctival surface, the transpalpebral method is indicated because the closed conjunctival sac formed in this technique serves to prevent orbital contamination during surgery [4]. When performing a transpalpebral enucleation, care must be taken during dissection not to open the conjunctival sac as this may increase the risk of contamination. However, even with precise dissection, a miniscule opening in the conjunctival sac is always made in the medial canthus when the nasolacrimal system is transected. Therefore, based on the National Research Council’s wound classification [5], enucleation can be considered a clean-contaminated procedure and perioperative antimicrobial prophylaxis is usually administered as a standard procedure.

Transpalpebral enucleation is often performed with the horse anaesthetized and in lateral recumbency, but a standing procedure has also been described [6, 7]. The standing procedure eliminates the expense and risk of general anaesthesia, but asepsis may be more difficult to maintain, which may increase the risk of surgical site infection.

Enucleation may lead to a deep indentation of the skin over the orbit, and implantation of an orbital prosthesis is a safe and inexpensive method in horses for improving cosmetic appearance after enucleation [8]. However, an implant serves as foreign material, which can be a nidus for infection and may allow biofilm formation [9, 10]. During clean-contaminated surgery, using an implant may increase the risk of surgical site infection (SSI). Unfortunately, in the advent of an SSI the implant will almost always be lost. In one study, a failure rate of 10.7% (3/28) is reported for silicone orbital implants after enucleation in the horse [8]. Therefore, the cosmetic advantage of an implant must be weighed against the disadvantage of an increased risk of SSI.

The aim of this retrospective study was to collect data on the risk factors for SSI and on long term follow-up (cosmetic results and return to work) following transpalpebral enucleation, to better inform clientele on the best surgical option for their horse.

## Methods

### Study design

Records of all horses undergoing transpalpebral enucleation at the Utrecht University’s Equine Clinic between 1 May 2007 and 1 October 2014 were reviewed. Data retrieved from case records included indication for enucleation, which eye was enucleated, the position of the horse during surgery (recumbent or standing), the cosmetic approach used (use of an implant or no implant), surgical findings (opening of the conjunctival sac and surgery time) and postoperative findings (development of SSI and duration of antimicrobial use).

**Fig. 1:**
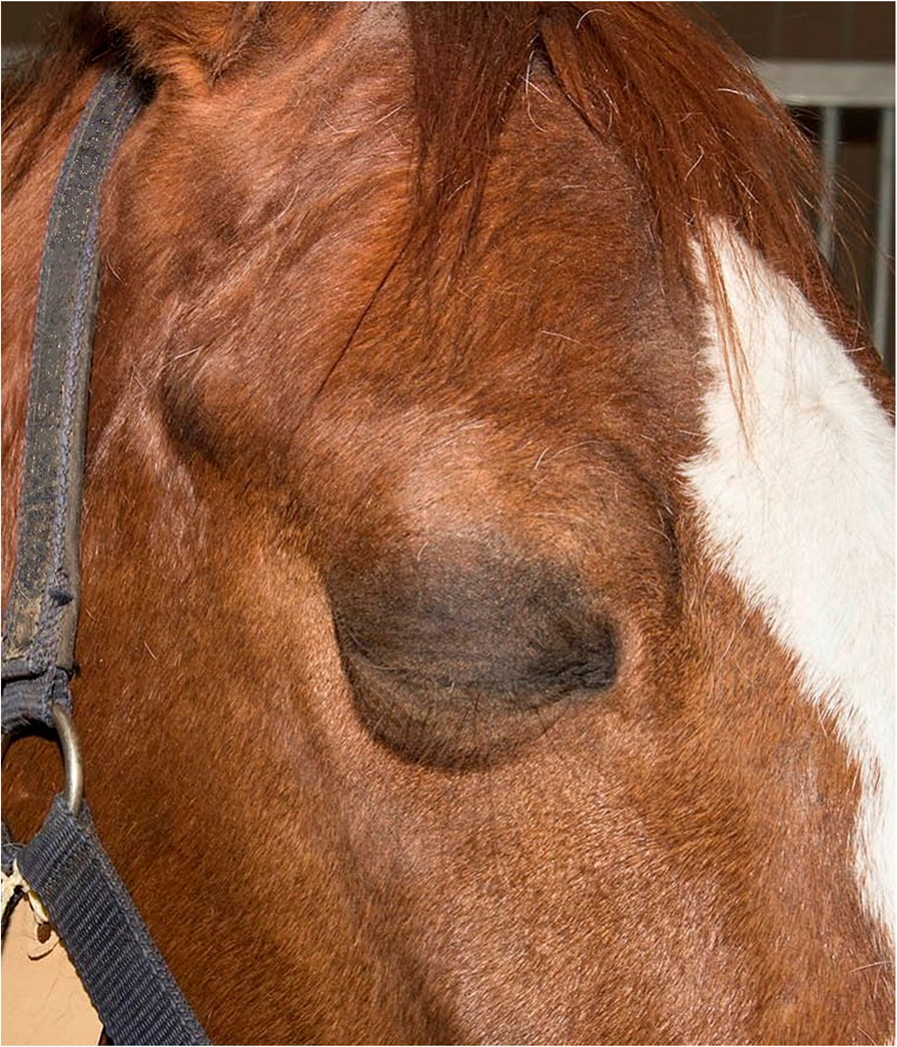
Image of a horse several months after enucleation with implant and an excellent cosmetic appearance

Follow-up information (no implant >30 days of follow up; implant >1 year of follow-up) from the owners’ evaluation forms or a telephone interview with the owner was used to retrieve long-term information about the development of an SSI. A telephone interview (>1 year after surgery) was used to determine the pre- and post-operative use and the performance ability of the horse and to evaluate the owners’ opinion about the final cosmetic appearance of the implant. Owners were asked to rate the cosmetic outcome as excellent, good or poor, with excellent for perfectly fitting implants (appearance identical to a closed contralateral eye; Figure 1), good for an implant showing minimal eyelid sinkage around the implant and poor for too large or too small implants.

Indications for enucleation were grouped into three groups. Group 1 (clean) included equine recurrent uveitis (ERU), too small globes (phthisis bulbi, microphthalmia) or too large globes (glaucoma, buphthalmos), and intraocular tumours. Group 2 (non-clean) included corneal perforation and rupture and infected ulcers. Group 3 (tumour) included extraocular tumours (including squamous cell carcinoma).

Wounds were classified as ‘infected’ when persistent serohaemorrhagic or purulent drainage from the surgical wound was observed by the surgeon or owner. Based on the Centers for Disease Control and Prevention (CDC) definitions, a deep or superficial SSI must have an onset within thirty days after operation or within one year if the operation included placement of an implant [5]. Therefore, when no implant was used, infection within thirty days after operation was considered an SSI and, when an implant was used, an infection up to one year after surgery was considered an SSI. Wounds were classified as ‘not infected’ if there were either no wound complications or (sero)haemorrhagic exudation was only observed within 24 h postoperatively.

### Surgical procedure of transpalpebral enucleation

Medical perioperative treatments were similar for all horses. Flunixin meglumine (1.1 mg/kg bwt, IV) was administered preoperatively. Prophylactic antibiotics (ampicillin (15 mg/kg bwt) or a combination of benzylpenicillin (40.000 IU/kg bwt) and gentamicin (6.6 mg/kg bwt)) were administered intravenously just before surgery, and all horses received a single intramuscular injection of procaine penicillin (22.000 IU/kg bwt) immediately after surgery. A longer, additional postoperative analgesic (meloxicam, 0,6 mg/kg bwt, PO) or antibiotic treatment was only provided to horses demonstrating signs of pain, postoperative swelling, or fever.

Surgeries were performed under general anaesthesia in lateral recumbency or standing with sedation and local analgesia. All horses received a retrobulbar block and, for horses undergoing a standing procedure, additional nerve blocks were performed as described in Pollock et al. [6]. A standard transpalpebral enucleation was performed [1] and the same surgical technique was used for the standing and the recumbent procedures. Preoperatively the surgical site was clipped and aseptically prepared, including flushing the lacrimal duct and the conjunctival sac with a mild disinfectant (0.1% povidone-iodine in saline). The eyelids were sutured together with a 2–0 non- absorbable monofilament suture in a continuous fashion. After closing the eyelids, the surgical site was again aseptically prepared and the transpalpebral enucleation was performed. Briefly, a full-thickness skin incision was created around the eyelid margins and a blunt and sharp subcutaneous dissection was performed posteriorly. After transecting the medial and lateral canthal ligaments, blunt dissection was continued along the sclera and the extraocular muscles and the optic nerve were transected. Before surgery the owners were always informed about the option of placing an orbital implant into the orbit to improve the cosmetic outcome. If preferred by the owner, after removal of the eye, a spherical silicone implant with a diameter dependent on the size of the orbit, was placed in the orbit (spherical silicone implants from Jardon eye prosthetics^1^). No preoperative or intraoperative measurements of the globe were performed to determine its size for selection of the implant. The optimal implant size was determined during surgery by fitting implants of different sizes into the orbit. The largest diameter size that could be fitted into the orbit without resistance - created by the size of the bony orbital rim - was chosen. Wounds were always closed completely, but the suture technique and material were at the surgeon’s discretion. No sutures were placed over the orbital implant to secure it in the orbital rim. When an implant was used a two layered closure was performed over the implant. The subcutaneous tissues of upper and lower eyelids were apposed using polygalactin 910 (Vicryl; Ethicon) or poliglecaprone 25 (Monocryl; Ethicon) in a simple continuous pattern. The skin was apposed with monofilament suture material; a continuous intradermal suture pattern (poliglecaprone 25) was used or a perforating simple interrupted pattern (poliglecaprone 25 or nylon (Ethilon; Ethicon)) was used. When no implant was used subcutaneous tissues and skin were closed using a two layered method - as described above -, or an interrupted perforating pattern was used for concurrent closure of the skin and subcutis (interrupted vertical mattress and simple interrupted pattern) using poliglecaprone 25 or nylon. If non-absorbable sutures or perforating patterns were used suture removal was performed 10–12 days after surgery. After surgery, wounds were covered with an adhesive bandage for recovery. The adhesive bandage was removed within 24 h after recovery and no (stent) bandage was used to cover the wound after this period.

### Data analysis

Variables analysed in this study are all categorical and are described in Table 1.

**Table 1:** The statistical variables included in the study

Variables:	
Indication	Group 1 (clean)
	Group 2 (non-clean)
	Group 3 (tumour)
Position of the horse	Recumbent
	Standing
Implant use	No implant
	Implant
Antibiotic treatment	Short term (24 h)
	Long term (>24 h)
Duration of the surgery	<mean surgery time
	>mean surgery time
Conjunctival sac	Not opened
	Opened

Univariable binary logistic regression was used to screen all variables for their association with the outcome measure ‘SSI’ vs ‘no SSI’. Multivariable binary logistic regression testing was performed with all variables and a manual backward elimination procedure was performed to fit a multivariable model. Non-significant variables were eliminated if their elimination did not affect the log OR of the significant variables more than 15%. Odds ratios and confidence intervals were determined for all variables. Due to the small numbers of SSIs no interaction between variables was tested. All statistical tests were performed using SPSS statistical software (SPSS 22). Statistical significance was set at *P* ≤ 0.05.

## Results

One hundred and seven cases of transpalpebral enucleation with primary closure and available follow-up about the development of an SSI were identified. Fifty-eight left eyes and 46 right eyes were removed. Eleven horses were operated on while standing and 96 horses were operated in lateral recumbency. Forty-nine horses had an implant used. The mean surgery time was 59 ± 22 min (range 23–123 min). No significant difference was found between the mean surgery time of recumbent (58 ± 21 min) and standing (72 ± 29 min) surgery (independent t-test *P* = 0.1). Enucleation was performed for a variety of reasons and, based on the previously described classification, 58 horses had a Group 1 indication (clean eye), 31 horses had a Group 2 indication (non-clean eye) and 18 horses had a Group 3 indication (tumour). Ninety-eight horses received a single dose of preoperative antibiotics (short term antibiotic treatment) and nine horses received prolonged antibiotic treatment (long term antibiotic treatment). Mean long term antibiotic treatment was 6.8 days (range, 3–18 days). Antibiotic treatment was prolonged for various reasons; in 2 cases antibiotic treatment was prolonged because perforation of the conjunctival sac occurred during surgery and increased contamination was considered likely. In 7 cases treatment was prolonged because of post-operative findings (postoperative swelling or fever) that might predispose to compromised wound healing. In two of these cases infection occurred (in one horse with an implant infection was presumed based on clinical signs, and in one horse without an implant infection was confirmed with culture), and an attempt was made to prevent implant loss by long-term antibiotic therapy. Neither infections resolved during antibiotic therapy and when clear signs of a sustained infection were apparent, antibiotic treatment was stopped and wounds were opened (with implant removal in the case with an implant) to allow sufficient drainage which resolved the infection quickly without negative consequences.

### Surgical site infections

Based on our inclusion criteria of infection there were 8 SSIs, resulting in an overall infection rate of 7.5% (8/107). Three infections were noted within 10 days after surgery, 3 between 10 days and 1 month, and 2 infections were noted between 1 and 6 months. In 4 cases of SSI, samples for culture were taken and they were positive for *S. equi zooepidemicus* (*n* = 2), *S. aureus* (*n* = 1), and several anaerobes (*n* = 1). Two owners reported late postoperative infection with purulent drainage and loss of the implant (3 and 4 years after surgery). In one case a sample for culture was taken which was positive for *S. equi zooepidemicus*. Based on the CDC definitions, these cases were not rated as SSIs. Including these two cases, the overall long-term (1–8 years follow-up after surgery) loss of an implant due to infection was 14.3% (7/49).

The incidence of SSIs in the different patient categories and their univariable screening for their association with the outcome measure ‘SSI’ vs ‘no SSI’ are outlined in Table 2.

**Table 2:** Percentage of SSIs for the different patient categories

	no SSI	SSI	Total	OR^a^	95% confidence interval^a^		Sig^a^
					Lower	Upper	
Indication							
Group 1: clean eye	55 (94.8%)	3 (5.2%)	58 (54.2%)	1			0.6^b^
Group 2: non-clean eye	28 (90.3%)	3 (9.7%)	31 (29%)	2	0.4	10.4	0.4
Group 3: tumour	16 (88.9%)	2 (11.1%)	18 (16.8%)	2.3	0.4	14.9	0.4
Total	99 (92.5%)	8 (7.5%)	107 (100%)				
Implant use							
No implant	55 (94.8%)	3 (5.2%)	58 (54.2%)	1			
Implant	44 (89.8%)	5 (10.2%)	49 (45.8%)	2.1	0.5	9.2	0.3
Total	99 (92.5%)	8 (7.5%)	107 (100%)				
Position of the horse							
Recumbent	90 (93.8%)	6 (6.3%)	96 (89.7%)	1			
Standing	9 (81.8%)	2 (18.2%)	11 (10.3%)	3.3	0.6	19	0.2
Total	99 (92.5%)	8 (7.5%)	107 (100%)				
Antibiotic treatment							
Short term	92 (93.9%)	6 (6.1%)	98 (91.6%)	1			
Long term	7 (77.8%)	2 (22.2%)	9 (8.4%)	4.4	0.7	25.9	0.1
Total	99 (92.5%)	8 (7.5%)	107 (100%)				
Surgery time							
< mean surgery time	55 (91.7%)	5 (8.3%)	60 (56.1%)	1.3	0.3	5.9	0.7
> mean surgery time	44 (93.6%)	3 (6.4%)	47 (43.9%)	1			
Total	99 (92.5%)	8 (7.5%)	107 (100%)				
Conjunctival sac							
Not opened	80 (93%)	6 (7%)	86 (80.4%)	1			
Opened	19 (90.5%)	2 (9.5%)	21 (19.6%)	1.4	0.3	7.5	0.7
Total	99 (92.5%)	8 (7.5%)	107 (100%)				

^a^Values are from the univariable logistic regression model

^b^Overall significance for indication

The following non-significant differences in percentages of SSI were found within the groups: horses with implants had a higher risk of SSI than those without implants (OR 2.1); horses in the non-clean eyes group (Group 2) and tumour group (group 3) had a higher risk of SSI than the horses in the clean eyes group (Group 1) (OR 2 (group 2) and 2.3 (group 3)); horses that underwent surgery in a standing position had a higher risk of SSI than those who were operated in a recumbent position (OR 3.3); horses that underwent prolonged antibiotic use had a higher risk of SSI than those that had short term antibiotic use (OR 4.4); horses with surgery time that was shorter than the mean surgery time had a higher risk of SSI than horses that had longer surgery times (OR 1.3); and finally, horses with surgeries in which the conjunctival sac was opened had a higher risk of SSI than horses that underwent surgeries in which the conjunctival sac remained closed during surgery (OR 1.4).

As shown in Table 3, horses in Group 1 had a significantly higher percentage of horses that received an implant compared with horses in the other groups (Fisher’s Exact Test *P* < 0.001).

**Table 3:** Distribution of implants over the groups (by indication)

	No implant	Implant	Total (*P* < 0.001^*^)
Indication			
Group 1: clean eye	21 (36.2%)	37 (63.8%)	58 (54.2%)
Group 2: non-clean eye	22 (71%)	9 (29%)	31 (29%)
Group 3: tumour	15 (83.3%)	3 (16.7%)	18 (16.8%)
Total	58 (54.2%)	49 (45.8%)	107 (100%)

* *P*-value from Fisher’s exact test

Therefore, multivariable logistic regression testing was performed with all variables in Table 2 to determine their association with the outcome measure ‘SSI’ vs ‘no SSI’ followed by a manual backward elimination procedure of nonsignificant variables. Table 4 presents the 3 variables found to be associated with SSI in the multivariable model (‘indication’ was a confounder and was kept in the model).

**Table 4:** Based on a multivariable logistic regression model, variables found to be associated with the likelihood of a horse having an SSI after enucleation

	OR	95% Confidence interval		Sig.
		Lower	Upper	
Indication				
Group 1: clean eye	1			0.1^a^
Group 2: non-clean eye	4.9	0.8	31.1	0.09
Group 3: tumour	5.9	0.7	49.4	0.1
Implant use				
No implant	1			
Implant	7.5	1.1	51.4	0.04
Position of the horse				
Recumbent	1			
Standing	12.1	1.3	112.9	0.03

^a^Overall significance for indication

The use of an implant (OR 7.5; *P* = 0.04) and a standing surgical procedure (OR 12.1; *P* = 0.03) were significantly associated with the development of SSI. Horses in Groups 2 and 3 trended towards being at a higher risk for developing an SSI (OR 4.9; *P* = 0.09 and OR 5.9; *P* = 0.1). Prolonged use of antimicrobials, the length of the surgery and whether the conjunctival sac was opened during dissection did not show a significant association with the risk of developing an SSI.

### Final cosmetic appearance of the implant

Long term follow-up on the final cosmetic appearance of the implant was obtained for 40 horses (in 4 horses long term follow-up about cosmetic appearance was not available and 5 horses lost their implant within 1 year after surgery because of a surgical site infection). Thirty-eight (95%) owners reported excellent to good cosmetic results. Two owners (5%) reported poor cosmetic outcome. Overall 97.5% (39/40) of the owners reported that in a similar case they would again opt for an implant.

### Return to work after unilateral enucleation

Long term follow-up information about pre- and post-operative use of 95 horses was available for evaluation. Based on pre-surgical discipline, the number of horses returning to their previous use was evaluated and the results are shown in Table 5.

**Table 5:** Return to work after unilateral enucleation, grouped on preoperative discipline

		Performance		Total
		Return	No return	
Discipline	Recreational/pleasure−/trail riding	52 (98%)	1 (2%)	53 (56%)
	Sport horse (dressage)	14 (100%)	0 (0%)	14 (15%)
	Sport horse (jumping)	4 (100%)	0 (0%)	4 (4%)
	Sport horse (harness)	4 (80%)	1 (20%)	5 (5%)
	Harness horse (pleasure)	5 (100%)	0 (0%)	5 (5%)
	Riding school horse (group lessons)	3 (100%)	0 (0%)	3 (3%)
	Broodmare	2 (100%)	0 (0%)	2 (2%)
	Retired	9 (100%)	0 (0%)	9 (10%)
Total		93 (97.9%)	2 (2.1%)	95 (100%)

Ninety-eight percent (84/86) of the horses returned to their pre-operative performance after enucleation. Two owners reported that their horses were not able to return to their preoperative use due to behavioural changes related to monocular vision; one horse did not return to work as a sport horse (harness), and one did not return to work as a pleasure horse. Nine horses were already retired at the time of the surgery so it was not possible to qualify their postoperative performance ability, but the owners reported that these horses had no difficulties in adapting to their life after enucleation.

## Discussion

The prevention of SSIs is a major goal during surgery, and proper surgical technique such as effective hemostasis, eradication of dead space and minimalizing wound contamination can reduce the risk of infection [11]. In this respect, some challenges are encountered during transpalpebral enucleation. Maintaining strict aseptic techniques is impaired because a small opening in the conjunctival sac is always made in the medial canthus when the nasolacrimal system is transected. Complete and effective haemostasis or eradication of dead space is not achievable during enucleation; although transecting the extraocular muscles at their tendinous insertions minimizes haemorrhage [1], removal of the eye - without subsequent placement of an orbital implant - always leaves a dead space that inevitably fills with blood. In this respect, dead space is reduced by placing an implant which might confound its predisposing factor for infection. The surgical site can be closed in three layers, and the orbital septum can be closed to secure the implant and to assist with haemostasis [1]. Although one can argue that this technique can help to reduce dead space, in the cases evaluated in this retrospective study no attempt was made to do so. These predisposing factors related to the technique of enucleation might have contributed to an overall infection rate of 7.5% in this study. Compared to Group 1, horses in Groups 2 or 3 (non-clean or tumour) had a considerably higher percentage of SSIs (9.7% and 11.1%, respectively). In Group 2, although the eyelids were sutured together before final aseptic preparation of the surgical site, minimal leakage from the conjunctival sac might have occurred during surgery causing an elevated concentration of microorganisms per gram tissue and increasing the risk of SSI. In Group 3, the intention to remove a tumour completely might have necessitated more extensive dissection, causing more tissue trauma, an increased inflammatory response and a larger dead space, which may explain the increased risk of SSIs.

Preoperatively, owners were always informed about the option of placing an orbital implant into the orbit to improve the cosmetic outcome, but they were discouraged from placing an orbital implant when there was evidence of neoplasia or infection (horses in Groups 2 and 3) [12]. The final decision about implant use was based on the owner’s preference, but as a result of our pre-operative advice, in Group 1, a significantly higher percentage of horses received an implant than in both of the other groups, which influenced the overall infection rate in Group 1. In Group 1, where no implant was used, no SSIs developed (0/21). In Group 1, horses that received implants 8.1% (3/37) developed an SSI.

Because transpalpebral enucleation is considered a clean-contaminated surgery, a small degree of contamination can be expected. In surgical wounds a number of microorganisms greater than 10^5^ per gram of tissue increases the risk of SSIs [13]. In the presence of foreign material the amount of contamination necessary for infection is markedly reduced [14]. Horses in groups 2 and 3 trended towards an increased risk of SSIs. Although implants can be used for horses that fall into one of these two groups, 17.6% (3/12) of the horses that did have implants used developed an SSI with loss of the implant.

The number of standing enucleations is small (*n* = 11) so no firm conclusions can be drawn. In the study of Pollock et al. (2008) evaluating 40 standing enucleations, 1 out of 32 horses (3.1%) with long term follow-up had serosanguinous discharge from the incision for 3 days [6]. In their study, an implant was used after standing enucleation in 4 out of 40 horses, but eight horses were lost to long-term follow-up and it was not further specified how many of them had an implant. Compared to this study we found a considerably higher infection rate of 18.2% for standing enucleation. In our study, the significant increase of SSIs in the standing enucleations compared to enucleations performed under general anaesthesia, may be explained by the difficulty in preventing contamination if the horse moves its head. It suggests that when implant placement is intended, an enucleation under general anaesthesia is preferable. Out of 11 standing enucleations performed in our study, in only one case was an implant used and in this case, the horse developed an SSI.

In one horse enucleation was started as a standing procedure, but due to continuous non-cooperative behaviour of the horse during closure of the eyelids - even after additional sedation and local analgesia - the procedure was eventually performed under general anaesthesia. This implies that character of the horse can also influence the final decision about performing enucleation with the horse standing or under general anaesthesia. One study about complications associated with anaesthesia for ocular surgery found that these horses were at greater risk of unsatisfactory recoveries [15]. In this respect, the advantage of avoiding the expenses and risks of general anaesthesia when performing a standing enucleation should also be taken into account.

The effect of accidentally opening the conjunctival sac on the percentage of SSIs is limited (OR 1.4) and shows that even when the conjunctival sac is opened an implant can be used. Eleven percent (1/9) of the horse in which the conjunctival sac was opened and an implant was used, developed an SSI. Eight percent (1/12) of the horses in which the conjunctival sac was opened developed an SSI when no implant was used. Surgeries were all performed after thorough flushing of the conjunctival sac and nasolacrimal duct with a mild disinfectant and with peri-operative antimicrobial prophylaxis. In two cases the post-operative antibiotic treatment was preventively prolonged after the conjunctival sac had been opened. These actions might have contributed to the limited deleterious effect of inadvertently opening the conjunctival sac during surgery.

A higher rate of SSI was found in horses that received prolonged antibiotic treatment, but this probably reflects the fact that prolonged antibiotic treatment was used in cases where infection was more likely. In 2 cases antibiotic treatment was prolonged because perforation of the conjunctival sac occurred during surgery and increased contamination was considered likely. In 7 cases treatment was prolonged because of post-operative findings (postoperative swelling or fever) lead us to anticipate possible compromised wound healing.

Apart from the 8 SSIs classified in this study, two late infections (after 3 and 4 years, respectively) were reported in the group with implants. Out of all horses with their implants in place after one year (no SSI; *n* = 44), in two horses (4.5%) late infection and loss/removal of the implant occurred. In both cases acute swelling of the orbital region occurred, followed by purulent drainage. According to the Centers for Disease Control and Prevention (CDC) definitions, an SSI must have an onset within one year if the operation included placement of an implant [5]. For the infections that occurred after 3 and 4 years, it is hard to imagine that the infection was there all that time in a subclinical form and these cases do not meet the criteria of an SSI. The surface of the implant may be a site of lower resistance where haematogenic bacteria have a better chance of producing an infection at a later stage. An infection of the implant might also be acquired through neighbouring tissue, after local trauma of the skin covering a slightly bulging implant at the nonvisual side. In the follow-up of horses that underwent enucleation without an implant, no late infection was reported.

Thirty-eight (95%) owners reported excellent to good cosmetic results. In our study, cosmetic outcome was reviewed by the owner and no objective postoperative measurement or standardized evaluation by the veterinary surgeon was performed. Although this would have been a more accurate way to rate cosmesis, achieving owner satisfaction about the cosmetic result is the most important goal in cosmetic procedures. In a study comparing enucleation with and without the use of a suture meshwork implant, 68% of the owners rated the cosmetic appearance after the use of an implant as ‘bad’, with a marked or deep sunk-in appearance [16]. In our study, only 2 owners reported poor cosmetic outcome because of the suboptimal size of the implants. One owner reported that the implant was too large and that the protruding implant led to frequent trauma of the skin (the horse showed late infection with loss of the implant 4 years postoperatively). The second owner reported that the implant was too small. In this case the spherical implant did not occupy the complete orbital volume and during the healing process, sinkage of the eyelids occurred and the implant within an orbital delineation was clearly visible under the skin. Due to the favourable cosmetic outcome of the spherical implants, it is advised to place this implant after transpalpebral enucleation if cosmetic appearance is important to the owner. If the owner is primarily concerned with the welfare of the horse, no implant should be used because cosmetic appearance is not of interest for the horse and increases the risk of SSI.

Since no preoperative or intraoperative measurements of the globe were performed to determine the size of the implant, fitting implants of different sizes into the orbit during surgery was needed to determine the optimal implant size. In our experience and based on high owner satisfaction about the cosmetic outcome, this is an effective method to determine the optimal size.

Two percent (2/86) of the horses failed to return to their pre-operative performance after enucleation due to behavioural changes related to monocular vision. A study reviewing 34 horses of different breeds and a variety of pre-surgical occupations, reported that 12% failed to return to work following unilateral enucleation for reasons related to monocular vision [17]. A large percentage of the population in that study were racing horses (10/34) and 20% of them were not returned to work. The population of horses in our study included no racing horses and this can be an explanation for the differences found. Independent of the differences in populations, our results confirm the conclusion that horses are able to return to a variety of occupations after unilateral enucleation.

Because this study was retrospective, several limitations were encountered. Since in some horses clinical signs did occur after the hospitalization period we lack samples for culture in 4/8 cases. Therefore, the classification of infection was limited to persistent serohaemorrhagic or purulent drainage. In 4/8 cases a sample for culture was taken and all samples confirmed infection. In 4/8 cases no culture or cytology was performed and in these cases non-infectious wound discharge and dehiscence or implant rejection can’t be excluded. As a consequence of how we did define infection some of the cases reported herein as infected, may not have been infected. This might have overrated the actual overall infection rate. Because of the single-centred nature of this study a relatively small number of horses were evaluated, but consequently no potential interhospital variations were applicable. Between 2007 and 2014 no temporal changes in the approach to patients or clients occurred in our hospital that we are aware of. Because we only started using the standing technique in 2011, the standing and recumbent surgeries are not equally distributed over time. In our clinic a standardized approach is employed by the veterinary technicians regarding aseptic preparation of the surgical patient and surgical instrumentation. These protocols are identical for all patients and were applied during the entire study period. Although all surgeons follow standardized guidelines regarding aseptic techniques employed, limitations such as the lack of complete similarity in surgical aseptic preparation (e.g. gloving technique) between surgeons were encountered. Between 2007 and 2014 a large variety of surgeons (*n* = 14) did perform the surgeries and none of the surgeons did the majority of the cases. Therefore, the results were not controlled for different surgeons and aseptic techniques employed. Even with these limitations valuable information to better inform the owners was obtained.

## Conclusion

Based upon this report the risk of SSI after enucleation is low in clean eyes and when no implant is used. Placing an implant and performing a standing enucleation significantly increases the risk of SSI. Enucleations performed in non-clean eyes and eyes with an extraocular tumour show a trend towards increased risk of SSI. Although implants can be used in these groups, 17% developed an SSI with loss of the implant. Owners should be informed about these risks so that they can make an informed decision about the choice of technique. If owners prioritize quick, uncomplicated healing above the final cosmetic outcome, an implant should not be placed. If final cosmetic outcome with implant placement is important to the owner, then an enucleation under general anaesthesia may be preferable.

Placing a silicone implant during enucleation is a simple and affordable technique to improve cosmesis and a high owner satisfaction about the cosmetic outcome is achieved.

Horses are able to return to a variety of occupations after unilateral enucleation.

## Abbreviations

CDC: Centers for Disease Control and Prevention
SSI: Surgical site infection

## Footnotes

1 Jardon Eye Prosthetics, Inc. Southfield, USA
